# Supplementary material for: Community-based surveillance system structures and knowledge level of community-based surveillance volunteers on priority diseases in Ghana
Source: PLOS Glob Public Health. 2025 Apr 16;5(4):e0003779. doi: 10.1371/journal.pgph.0003779 (PMC12013933; doi:10.1371/journal.pgph.0003779)
Supplement: Attachment — Submitted filename: CBS Responses to Reviewers Comments 08092024 (1).pdf [file pgph.0003779.s002.pdf]

Responses to Reviewers Comments (Reviewer 1)

Topic Community-based System Structures and Knowledge level of CBSVs on Priority Diseases and Events in Ghana

The authors would like to appreciate the reviewer for the remarks and the expert review in improving the quality of our work. These are our responses to the reviewer. In this document, we provide a point-by-point response to the comments and suggestions of the reviewers. Our responses are in the tabular form. The corrections in the main work have highlighted yellow.

Reviewer 1

**Topic**	**Reviewer's comments**	**Authors Response**	**Page/Remarks**
**Abstract**			
Methodology	Specify how knowledge assessment was conducted	A brief on how the assessment of the CBSVs was done has been stated in the abstract. Detailed description has been done in the work	See abstract section, under methods, line 3&4.<br>Also, see page 5, para 2 under data collection techniques, line 5&6<br>Detailed explanation done at page 6, paragraph 2&3 under data processing and analysis
Results	Provide concrete data to support low knowledge.	Concrete data have been stated in percentages and have been compared with the national standards	See result section under abstract, lines 2, 3, 4, 5 and 6
Conclusion/ recommendations	The conclusion that the surveillance system is weak has been overs simplified.<br>Provide actionable recommendations	The statement was made after the key findings were compared with the national standard of 80% or more. The implications of the findings also warrant that statement. Actionable conclusions and recommendations have been provided.	See result section under abstract, lines 2, 3, 4, 5 and 6<br><br>See also the recommendation section under abstract.
**Introduction**	State in the introduction the problem the research will address, give global	Problem the research will address have been stated in the introduction.	See page 3, the last paragraph.

	and regional overview. Why the need for the research, provide potential factors that contribute to lack of active surveillance	Global and regional overview and perspective stated in the introduction<br>Literature reviewed on system structures and knowledge level on priority diseases<br>Factors for lack of active surveillances	See page 1, paragraphs 1&2.<br><br>See page 1, paragraph 3&4<br><br>See page 3, last paragraph, lines 3&4

**Topic Methodology**	**Reviewer's Comments**	**Authors Response**	**Page/Remarks**
Study design	Provide rationale for using a cross-sectional study for the research	Rationale stated.<br>Strengths and weaknesses stated	See page 4 under study design line 1,2 2,<br>Line 4 and 5. strengths<br>Line 6 for the weaknesses
Study area	Explain why the Central Region was chosen for the study	The reason for selecting Central Region for the study has been stated	See page 4, para 1, line 10-15 under study area
Sampling	Provide details of sample size calculation	The details of the sampling calculation provided	See page 4, line 3 to 12 under sampling strategy
Data collection techniques	Describe steps in data collection,<br>Include details of questionnaire, pilot testing and how data quality was ensured. Provide details on training and how it ensured validity and reliability	Details of the questionnaire have been stated.<br>Pretest details stated.<br><br>How validity and reliability of the study were ensured have been stated in the manuscript.<br><br>Details of training contents provided	See last paragraph under data collection<br>See page 5, para 2, line 2 3, 4, 5. Under data collection<br>See page 5, para 3,<br><br>See page 5 para 1, lines 6, 7, 8. Also para 3 provide details<br>See contents under training of data collectors
Data Analysis	How was assumptions checked<br>Explore potential interactions between variables	Assumptions details provided<br>Analysis done based on the study objectives, potential interaction be done in subsequent articles	See page 7, last para under data analysis
Scoring	Use a table or diagram to visually represent the	Table has been used to explain scoring system	See page 7, Table 1.

	scoring system and interpretation criteria		
Ethics	Explain how participants were informed about the study, how consent were obtained, and measures taken to ensure confidentiality and data protection	Details of inform consent have been provided and measures taken to ensure confidentiality stated	See page 7, para 1, 6 to 15

**Topic**	**Reviewer's Comments**	**Authors Response**	**Page/Remarks**
**Results**			
	Table 5, Four categories collapsed into binary	Table 6 give details and could be referenced. Used binary to make interpretations to be more meaningful	See page 12, Table 6
**Discussion**	Compare findings with existing literature	Existing literature provided and finding compared and discussed	See page 14 and 15
conclusion	Lacks specificity	Based on the finding concise conclusion has been stated	See page 15
Recommendation	Consider including community participation and ownership	Community participation and ownership included in the recommendations	See page 16, under recommendation

Responses to Reviewers Comments (Reviewer 2)

**Topie**	**Reviewer's Comments**	**Authors Response**	**Page/Remarks**
	Deficient on priority disease highlights and the need for its knowledge among CBSVs	Priority diseases and events stated in the introduction.<br>Potential consequences for inadequate knowledge on	See page 2 para 4, line 3 to 7.<br><br>See paragraph 3, last 4 lines.

		priority diseases have also been stated	
	CHPS, DHMT and CHO first mentioned not explained.	These have been explained	See page 1, line 9 and 10 under recommendations<br>See page 5, last paragraph under data collection techniques
	Situate study objective at the last paragraph of the introduction	This has been stated	See page 3, the last 3 sentences under the introduction
	Chi-square test not appropriate for differences.	This has been checked	See page 2, last paragraph of data analysis
	Frequencies and percentages of respondents' occupation does not align in the report	These have been checked	See page 8, Table 2
	Title of the table 5 not align with what is in the report	Association and differences corrected to align	See page 12 now Table 6 and page 13 first paragraph under Table 6
	Fishers' exact notes should be corrected under 5	This has been corrected	See page 13, now Table 6
