# Supplementary material for: Community-based surveillance system structures and knowledge level of community-based surveillance volunteers on priority diseases in Ghana
Source: PLOS Glob Public Health. 2025 Apr 16;5(4):e0003779. doi: 10.1371/journal.pgph.0003779 (PMC12013933; doi:10.1371/journal.pgph.0003779)
Supplement: Attachment — Submitted filename: Responses to Reviewers Comments CBS study 27102024.pdf [file pgph.0003779.s003.pdf]

Responses to Reviewers Comments (Reviewer 1)

Topic Community-based System Structures and Knowledge level of CBSVs on Priority Diseases and Events in Ghana

The authors would like to express their appreciation to the reviewers for the remarks and expert review in improving the quality of our work.

The following are our responses to the reviewers. In this document, we have provided point-by-point responses to the comments and suggestions made by the reviewers. We have preceded the Reviewer's Comments with "RC" and the Authors' Responses "AR".

Reviewer 1

RC: Abstract: specify how knowledge assessment of CBSVs was conducted.

AR: Thank you for the comments. A brief on how the assessment of the CBSVs was done has been stated in the abstract (See abstract section, under methods, line 3&4). Detailed description has been done in the work (See page 6, para 2 under data collection techniques, line 8,9&10. Detailed explanation done at page 7, paragraph 2&3 under data processing and analysis).

RC: Results under abstract: stated CBSVs have low knowledge but does not provide concrete data to support the claim of low knowledge.

AR: This comment is well appreciated. A Concrete data have been stated in percentages and have been compared with the national standards (See result section under abstract, lines 2, 3, 4, 5 and 6).

RC: The conclusion that the surveillance system is weak has been overssimplified. Provide actionable recommendations.

AR: The author greatly appreciates this comment. The statement was made after the key findings were compared with the national standard of 80% or more. The implications of the findings also warrant that statement. Actionable conclusions and recommendations have been provided (See result section under abstract, lines 2, 3, 4, 5 and 6. See also the recommendation section under abstract).

RC: State in the introduction the problem the research will address, give global and regional overview. Why the need for the research, provide potential factors that contribute to lack of active surveillance.

AR: This comment is well noted with thanks. Problem the research will address have been stated in the introduction (See page 3, the last paragraph under introduction). Global and regional overview and perspective stated in the introduction (See page 1, paragraphs 1&2 under introduction). Literature reviewed on system structures and knowledge level on priority diseases (See page 2, paragraph 3&4 under introduction). Factors for lack of active surveillances (See page 3, last paragraph, lines 3&4).

RC: Provide rationale for using a cross-sectional study for the research and the advantages it offer.

AR: Thank you for this comment. The rationale has been stated, also the strengths and weaknesses stated. See page 4 under study design line 1,2, Line 4 and 5. See line 6 for the weaknesses.

RC: Explain why the Central Region was chosen for the study.

AR: This comment is well appreciated. The reason for selecting Central Region for the study has been stated. See page 4, paragraph 1 under study area, line 10-15.

RC: Provide details of sample size calculation.

AR: Thank you for this comment. The details of the sampling calculation provided. See page 4, line 3 to 12 under sampling strategy.

RC: Describe steps in data collection, include details of questionnaire, pilot testing and how data quality was ensured. Provide details on training and how it ensured validity and reliability.

AR: The author appreciates this comment. Details of the questionnaire have been stated (See last paragraph under data collection). Pretest details stated (See page 5, para 3, line 2 3, 4, 5-12, under data collection techniques). How validity and reliability of the study were ensured have been stated in the manuscript (See page 5, para 3). Details of training contents provided (See page 6 para 1, lines 3-6, under training of data collectors).

RC: How were assumptions checked. Explore potential interactions between variables.

AR: This comment is greatly appreciated. The assumptions details have been provided. Analysis was done based on the study objectives, however, potential interaction between variables would be done in subsequent articles (See page 6, last para under data analysis after Table 1).

RC: Use a table or diagram to visually represent the scoring system and interpretation criteria.

AR: Thank you for this comment. A table has been used to explain the scoring system (See page 7, Table 1).

RC: Explain how participants were informed about the study, how consent was obtained, and measures taken to ensure confidentiality and data protection.

AR: This comment well noted. Details of inform consent have been provided and measures taken to ensure confidentiality stated (See page 7, para 1 under ethics statement, lines 6 to 15).

RC: Table 5, Four categories collapsed into binary.

AR: Thank you for this comment. Table 6 gives details and could be referenced. However the author collapsed categories into binary to make interpretations to be more meaningful (See page 13, Table 6).

RC: Compare findings with existing literature.

AR: The author agrees with this comment with great appreciation. The existing literature has been provided and finding compared and discussed (See page 14 and 15).

RC: Conclusion lacks specificity

AR: Thank you for your comments. Based on the finding the author has stated concise conclusions (See page 15).

RC: Consider including community participation and ownership

AR: This comment is greatly appreciated. Community participation and ownership have been included in the recommendations (See page 16, under recommendation)

Reviewer 2

RC: introduction is deficient on priority disease highlights and the need for its knowledge among CBSVs.

AR: This comment is well noted. Priority diseases and events have been stated in the introduction (See page 2 para 4, line 3 to 7). Potential consequences for inadequate knowledge on priority diseases have also been stated (See paragraph 3 under introduction, last 4 lines).

RC: CHPS, DHMT and CHO first mentioned not explained.

AR: Thank you, this comment. These acronyms have been explained (See page 1, line 8 and 10 under recommendations. See page 5, last paragraph under data collection techniques).

RC: Situate study objective at the last paragraph of the introduction.

AR: This comment is well noted. The study objective has been stated in the last paragraph (See page 3, the last 3 sentences under the introduction).

RC: Chi-square test not appropriate for differences.

AR: This has been checked (See page 7, last paragraph of data analysis).

RC: Frequencies and percentages of respondents' occupation does not align in the report.

AR: Thank you for this comment. These corrections have been made (See page 8, Table 2).

RC: Title of the table 5 not align with what is in the report.

AR: I appreciate this comment. Association and differences corrected to align (See page 12 now Table 6 and page 13 first paragraph under Table 6).

RC: Fishers' exact notes should be corrected under Table 5.

AR: Thank you for this comment. This has been corrected (See page 13, now Table 6)
