# Supplementary material for: Community-based surveillance system structures and knowledge level of community-based surveillance volunteers on priority diseases in Ghana
Source: PLOS Glob Public Health. 2025 Apr 16;5(4):e0003779. doi: 10.1371/journal.pgph.0003779 (PMC12013933; doi:10.1371/journal.pgph.0003779)
Supplement: Attachment — Submitted filename: Response to Reviewers.pdf [file pgph.0003779.s004.pdf]

RESPONSES TO REVIEWERS'

Topic Community-based System Structures and Knowledge level of CBSVs on Priority Diseases and Events in Ghana

The authors would like to express their appreciation to the reviewers for the remarks and expert review in improving the quality of our work.

The following are our responses to the reviewers. In this document, we have provided point-by-point responses to the comments and suggestions made by the reviewers. We have preceded the Reviewer's Comments with "RC" and the Authors' Responses "AR".

RC: Please revise the abstract to 300 words in length (or as close as possible). The abstract should mention techniques used without going into methodological detail and should summarize the most important results. While I understand that some edits were made to the abstract to include suggestions from reviewers, particularly adding the recommendations section, unfortunately, it is necessary to reduce the abstract length. Please also note that subheadings are not used in the abstract, although conceptually the abstract is divided into three sections (Background, Methods/Principal findings, and Conclusions/Significance).

AR: Thank you for the comments. The abstract has been revised to 300 words in length. The sub-headings have been put into paragraphs to make it easier for readers to comprehend. (See page 1).

RC: The introduction should be proofread to correct some sense and typing errors, and to ensure that information is concisely presented. Some extraneous statements that are not directly related to the study (such as the citation on knowledge of meningitis by travelers from the UK to the meningitis belt) could also be removed.

AR: These comments are well appreciated. The introduction has been proofread, all typing errors have been corrected and extraneous statements have been removed. (See page 2-3)

RC: In the methods, please clarify what is meant by a systematic random sample, since these terms appear to be contradictory – sampling is usually either systematic or random.

AR: The author greatly appreciates this comment. The method of sampling has been stated as systematic sampling and briefly explained in a sentence. (See page 5, 2nd paragraph under data collection technique)

RC: In the methods, data analysis section, please add a sentence clarifying how you reclassified your level of knowledge from 4 classes to a binary outcome for the logistic regression.

AR: Thank you for this comment. A statement has been made to clarify how classification of the knowledge level was made into binary outcome. (See page 7, the last paragraph under data processing and analysis).

RC: Review reference list and ensure that it is complete and correct.

AR: The reference list has been reviewed and some wrongly stated numbers intext that were not part of the reference list have been deleted.
