# Supplementary material for: Community-based surveillance system structures and knowledge level of community-based surveillance volunteers on priority diseases in Ghana
Source: PLOS Glob Public Health. 2025 Apr 16;5(4):e0003779. doi: 10.1371/journal.pgph.0003779 (PMC12013933; doi:10.1371/journal.pgph.0003779)
Supplement: Attachment — Submitted filename: Response to Editors Comments.docx [file pgph.0003779.s001.docx]

## PLOS Global Public Health: Edits requested on your submission PGPH-D-24-01347 - [EMID:cf213c5d6d603224]

External

Inbox

Search for all messages with label Inbox

Remove label Inbox from this conversation

| PLOS Global Public Health <em@editorialmanager.com> | 12:15 PM (8 hours ago) |  |    |
| --- | --- | --- | --- |
| to me  | | |  |

PGPH-D-24-01347
Community-based Surveillance System Structures and Knowledge Level of Community-based Surveillance Volunteers on Priority Diseases in Ghana


Dear Dr. Hormenu,

We've checked your submission and before we can proceed, we need you to address the following issues:

1) Before we can continue with your submission, please upload a copy of the original ethics approval letter from the University
of Cape Coast’s Institutional Review Board in Cape Coast (ID: UCCIRB/CES/2019/45), and the Ghana Health Service Ethics Review Committee in Accra (ID: GHS-ERC001/02/20), as required by question 1 of the human subject research checklist. If the original letter is not in English, please also provide an English translated version in the supporting file. Please note that if this documentation is not included when your manuscript is resubmitted, it may be rejected. Thanks for this suggestion. We have added the ethical clearance from the two institutions

2) Please report in the Methods section the day, month and year of the start and end of the recruitment period for this study. Please note that if this information is not included when your manuscript is resubmitted, it may be rejected. The day, month and year has been added to the text in page 3.

We've returned your manuscript to your account. Please resolve these issues and resubmit your manuscript within 21 days. If you need more time, please email the journal office at [globalpubhealth@plos.org](mailto:globalpubhealth@plos.org). We are happy to grant extensions of up to one month past this due date. If we don't hear from you within 21 days, we will withdraw your manuscript.

Please log on to PLOS Editorial Manager at <https://www.editorialmanager.com/pgph/> to access your manuscript. You will find your manuscript in the 'Submissions Sent Back to Author' link under the New Submissions menu. Be sure to remove your previous manuscript file if you are uploading a new file in response to these requests. After you've made the changes requested above, please be sure to view and approve the revised PDF after rebuilding the PDF to complete the resubmission process.

We are requesting these changes to comply with the [globalpubhealth@plos.org](mailto:globalpubhealth@plos.org) submission guidelines (<https://journals.plos.org/globalpublichealth/s/submission-guidelines>). Please note that we won't send your manuscript for review until you have resolved the above requests.

Thank you for submitting your work to [globalpubhealth@plos.org](mailto:globalpubhealth@plos.org) and supporting our mission of Open Science.

Kind regards,

Jan Louis Adajo Non
PLOS Global Public Health
